# Rescue of the First Mitochondrial Membrane Carrier, the mPiC, by TAT-Mediated Protein Replacement Treatment

**DOI:** 10.3390/ijms26094379

**Published:** 2025-05-05

**Authors:** Samar Zabit, Orly Melloul, Michal Lichtenstein, Erin L. Seifert, Haya Lorberboum-Galski

**Affiliations:** 1 Department of Biochemistry and Molecular Biology, The Institute for Medical Research Israel-Canada (IMRIC), Faculty of Medicine, The Hebrew University of Jerusalem, Jerusalem 9190501, Israel; samar.zabit@mail.huji.ac.il (S.Z.); orly.elbaz@mail.huji.ac.il (O.M.); michallic@ekmd.huji.ac.il (M.L.); 2 MitoCare Center, Department of Pathology and Genomic Medicine, Thomas Jefferson University, Philadelphia, PA 19107, USA; erin.seifert@jefferson.edu

**Keywords:** mitochondria, mitochondrial phosphate carrier, mitochondrial phosphate carrier deficiency (MPCD), protein replacement treatment, TAT-mediated therapy

## Abstract

The mitochondrial phosphate carrier (mPiC), encoded by the nuclear gene *SLC25A3*, is synthesized with an N-terminus mitochondrial targeting sequence (MTS), enabling its import into the mitochondria. mPiC imports inorganic phosphate (P_i_) into the mitochondrial matrix for ATP production and other matrix phosphorylation reactions, as well as regulates mitochondrial Ca^2+^ uptake and buffering of matrix Ca^2+^. PiC also imports copper (Cu), crucial to COX subunit holoenzyme assembly. Variants in *SLC25A3* exist and lead to mPiC deficiency (MPCD), cause a rare autosomal recessive disease with no current cure; patients with MPCD usually die within the first year of life. We have developed a novel therapeutic approach using TAT-mPiC fusion protein for cellular delivery since the TAT peptide enables delivery of proteins across biological membranes. We designed, produced, and purified the TAT-mPiC fusion protein. The fusion protein is delivered into the mitochondria and localizes within the mIM, its natural cellular location, as a processed protein. Treatment of mPiC-knockdown cells with TAT-mPiC fusion protein increased cell growth and improved bioenergetic capabilities, as measured by oxygen consumption rate (OCR), ATP production, and reduction in lactate secretion. Most importantly, TAT-mPiC restored P_i_ and Cu delivery into the mitochondrial matrix. TAT-mPiC fusion protein also restored the mitochondrial activity of cells harboring various mitochondrial defects. This study presents the first successful delivery of a mitochondrial transmembrane carrier using the TAT-fusion system, offering a potential early treatment strategy for newborns with mPiC deficiency.

## 1. Introduction

The mitochondrial phosphate carrier (mPiC) is a mitochondrial inner membrane protein, encoded by the nuclear gene *SLC25A3* located on chromosome 12q23 [1,2], and is part of the mitochondrial carrier family (MCF/SLC25) proteins [3]. It has six transmembrane segments, 3-fold symmetry, and N and C termini projecting into the intermembrane space [1]. mPiC transports inorganic phosphate (P_i_) in symport with H^+^ or in antiport with OH^−1^. Earlier studies demonstrated that mPiC mRNA is alternatively spliced to produce two different variants, mPiC-A, expressed in the heart, skeletal muscle, and pancreas in high amounts, and mPiC-B, the predominant isoform in other tissues [2]. Isoforms A and B contain 362 and 361 amino acids, respectively, and they show different substrate affinities and transport rates [1].

mPiC is the primary inorganic-phosphate carrier, thereby enabling oxidative phosphorylation (OXPHOS) by the addition of P_i_ to ADP to yield ATP. P_i_ in the mitochondrial matrix can both stimulate the mitochondrial calcium uniporter (mtCU)-mediated Ca^2+^ influx and suppress the exchanger-mediated Ca^2+^ efflux, thus controlling mitochondrial Ca^2+^ levels by forming Ca_3_(PO_4_^−^)_2_ salts [4,5,6].

Recent studies have shown that mPiC also transports copper ions (Cu) through the mitochondrial membrane to the mitochondrial matrix, ultimately aiding in the metalation of cytochrome c oxidase (COX), the terminal electron accepting complex of the mitochondrial respiratory chain. The reduction of O_2_ is mediated by COX, where Cu is an essential component for electron transfer [7].

mPiC deficiency (MPCD) is a rare autosomal recessive disease characterized by the absence or mutation of mPiC (OMIM 610773) [8]. This disorder is characterized by onset of cardiorespiratory insufficiency soon after birth and may result in death in infancy. Most affected individuals exhibit hypotonia, delayed motor development, and exercise intolerance, though cognitive development is typically normal. Laboratory studies characteristically show increased serum lactate. Muscle biopsy shows abnormal mitochondria and lipid accumulation [9]. We have reviewed all reported cases of pathological *SLC25A3* variants [4]. To date, there are three different reported *SLC25A3* mutations, all leading to severe striated muscle pathology soon after birth. All reported cases are summarized in Table S1.

Treatment options for MPCD, as for other mitochondrial diseases, offer only symptomatic supportive care and include nutritional management, exercise and/or vitamin or amino acid supplements; thus, new strategies for treatment are greatly needed [10].

One such approach is enzyme/protein replacement therapy (E/PRT). E/PRT is a therapeutic strategy for treating metabolic diseases whereby the deficient protein is produced, mainly as a recombinant protein, purified and given to patients on a regular basis [11]. In the case of delivering a protein to the mitochondria, the fusion protein is generated with three main motifs: a cell-penetrating motif, which facilitates the translocation of the fusion protein into various cells and tissues; a mitochondrial targeting sequence, which enables the protein to be imported into the mitochondria; and the desirable human active protein motif, which replaces the defective mitochondrial protein.

Cell-penetrating peptides (CPPs) can deliver active enzymes across the cellular membrane and in particular across the blood-brain barrier (BBB), so facilitating the treatment of disorders involving the central nervous system (CNS). One such CPP is the trans-activator of transcription protein (TAT), an 11-amino acid (residues 47–57 (YGRKKRRQRRR)) arginine- and lysine-rich sequence of the Tat-protein encoded by HIV-1. TAT-cargoes are rapidly and efficiently internalized into cultured cells, intact tissues, and live tissues, while retaining their biological activity [12]. This characteristic makes this delivery system highly promising for the development of therapies, especially for mitochondrial diseases. In 2008, our group was the first to introduce this approach for treatment of a mitochondrial enzyme deficiency disorder [13]. We successfully used the TAT-delivery system fused with the human mitochondrial lipoamide dehydrogenase (LAD) enzyme to deliver a mitochondrial enzyme into the mitochondria [13]. The TAT-LAD fusion protein was tested in an in vivo model, where it was delivered into the E3-deficient mouse tissues, resulting in a significant increase in the enzymatic activity of the mitochondrial multienzyme complex pyruvate dehydrogenase complex within various tissues, and most importantly, the brain [14]. Following this, the TAT-delivery system was tested for NADH dehydrogenase (ubiquinone) complex I assembly factor 4 (NDUFAF4) [15], for frataxin using its native MTS or a heterologous MTS, the citrate synthase MTS [16], and for methylmalonyl-CoA mutase (MCM) [17].

In 2010, another group applied the TAT-mitochondrial protein machinery to deliver a complex IV assembly matrix protein involved in mitochondrial copper transfer, TAT-L-Sco2, a protein anchored to the mitochondrial inner membrane by a single helix. The protein was successfully delivered to the mitochondria both in vitro [18] and in vivo [19]. The same approach was used to deliver NADH-quinone internal oxidoreductase (Ndi1) [20], NADH dehydrogenase (ubiquinone) Fe–S protein (NDUFS8; a membrane interacting protein) [21], and an antioxidant protein metallothionein (MT) (for which TAT was fused to a short artificial MTS to produce CAMP) [22]. These studies confirmed that the concept of TAT-mediated E/PRT is a very promising approach to rescuing mitochondrial protein deficiencies, mainly for mitochondrial matrix proteins.

In the current study, we tested whether mPiC can be delivered to cells in the form of a TAT-fusion protein, as the first attempt to deliver a multi-pass transmembrane protein into mitochondria. We designed, produced, and purified the TAT-mPiC fusion protein. The fusion protein is delivered into the mitochondria and localizes within the mitochondrial inner membrane (mIM), its natural cellular location, as a processed protein. Treatment of mPiC-knockdown cells (mPiC-KD) with the TAT-mPiC fusion protein increased cell growth and improved bioenergetic capabilities, as measured by oxygen consumption rate (OCR) and reduction in lactate secretion. Most importantly, treatment with TAT-mPiC fusion protein restored the delivery of Pi and Cu into the mitochondria of deficient cells. Emphasizing the major role of inorganic phosphate in ATP production within the mitochondria, the basal OCR of another three mitochondrial protein defective cells was improved when treated with the TAT-mPiC fusion protein.

## 2. Results

### 2.1. Construction and Expression of the TAT-MTS-mPiC Fusion Proteins

**Figure 1:**
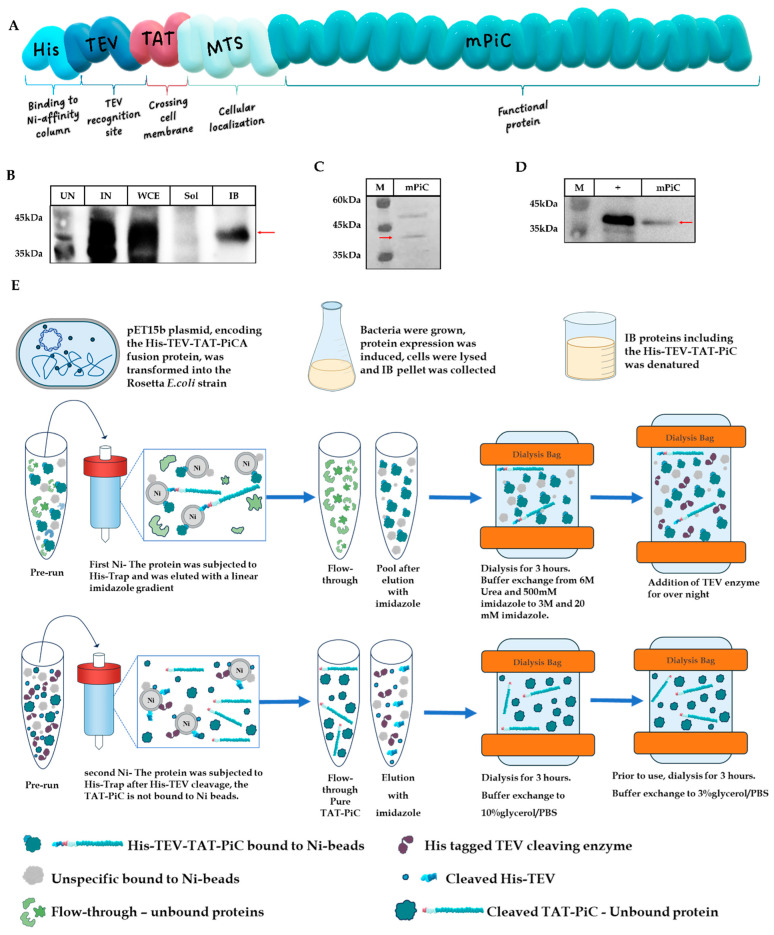
Construction and production of His-TEV-TAT-mPiC.A fusion protein: (**A**) Schematic presentation of the fusion protein, His-TEV-TAT-Phosphate carrier variant A (His-TEV-TAT-mPiC.A) MW = 44 kDa. Following construction, the chimeric protein was expressed in Rosetta strain *E. coli*. (**B**) Uninduced (UN), 0.5 mM IPTG induction (IN), whole cell extract (WCE), soluble (Sol) and inclusion bodies (IB) sub-fractions were separated on 12% SDS-PAGE and Western blot analysis with anti-PiC antibody (1:1000) was performed. Following expression, the fusion protein was purified using an affinity column and cleaved using TEV enzyme. The cleaved and purified protein was separated on 12% SDS-PAGE and analyzed using (**C**) Coomassie blue staining and (**D**) Western blot analysis with anti-PiC antibody (1:1000). (**E**) Schematic illustration of His-TEV-TAT-mPiC fusion protein transformation, sub-cellular fractionation and purification process culminating with the relatively purified TAT-mPiC fusion protein. Protein markers and positive control (semi purified TAT-mPiC) on gels are marked by M and +, respectively. Red arrows indicate the full length exogenous mPiC fusion protein. Light blue = His-tag for Ni column binding, Dark blue = TEV protease recognition sequence, Bordo = TAT sequence allowing the cargo to cross cell membranes, Mint green = MTS-mitochondrial targeting sequence allowing the protein to localize into mitochondria, Turquoise = the functional human mitochondrial Phosphate Carrier (mPiC) protein.

Our aim was to design mPiC as a fusion protein able to cross the cellular membranes, reach the mitochondria and localize into the mIM, thereby, at least partially rescuing the *SLC25A3* mutations that lead to drastically lower mPiC levels. We constructed the fusion protein TAT-mPiC (isoform A). The fusion protein includes a His-TEV sequence (His-tag and TEV recognition site for protein purification), a TAT sequence enabling it to cross the cellular membranes, the natural MTS sequence of mPiC for transport into the mitochondria, and the full sequence of the mature human mPiC. Figure 1A illustrates the schematic structure of the final TAT-mPiC fusion protein.

The fusion protein was expressed in a bacterial system, and the conditions for expression (host system, incubation temperature, IPTG-induction concentration, and time of induction) were optimized. The optimal conditions were: Rosetta *E. coli* strain as the expression system, growth at 37 °C and 0.5 mM IPTG for 4 h of induction; leading to the expression of the maximal amount of the TAT-mPiC fusion protein (M_w_ ≈ 44 kDa). Sodium dodecyl sulfate polyacrylamide gel electrophoresis (SDS-PAGE) analysis and Western blotting confirmed the identity of the fusion protein (Figure S1A,B).

Following expression, subcellular fractions of whole cell extract (WCE), soluble fraction (Sol) and inclusion bodies (IB) were prepared, separated on SDS-PAGE, and characterized by Western blot analyses, using both anti-PiC and anti-His antibodies. We found that the fusion protein mostly aggregated in the inclusion bodies (IB) of the bacterial expressing cells, as would be expected for a highly hydrophobic membrane protein and thus developed and calibrated a protocol for producing a biologically active fusion protein (Figure 1B).

### 2.2. Purification of the TAT-mPiC Fusion Protein

The IB fraction of bacterial expressing cells was separated and suspended in a denaturation buffer, containing 6 M urea. This denatured fusion protein was loaded onto a nickel-charged affinity column, under denaturing conditions, utilizing the His-tag on the N-terminus of the protein. Following proteins’ elution, Western blot analysis showed that the fusion protein was concentrated in four fractions. However, a parallel SDS-PAGE analysis revealed a very low purity of the fractions, due most probably to non-specific binding of other proteins. Consequently, we added another purification step utilizing the TEV recognition sequence, designed in our fusion protein. Peak fractions from the first affinity column were collected and briefly dialyzed with denaturation buffer of a lower urea concentration (3 M), for three reasons: 1. The fusion protein was still partially denatured leaving the N-termini exposed for the cleaving process; 2. A gradient decrease in the chaotropic denaturant was required to guarantee that the fusion protein will not aggregate again; 3. In 6 M urea, the TEV enzyme is fully denatured and thus inactive. Following the short dialysis step, the TEV enzyme was added to the same dialysis bag for overnight incubation, to get rid of the His-tag sequence. The solution was reloaded onto a nickel-charged affinity column, to increase the protein’s purity, collecting this time the flow-through fraction that contains the cleaved TAT-mPiC fusion protein (Figure S1C). This fraction was dialyzed against 10% glycerol/PBS (Figure 1C,D). The 10% glycerol/PBS buffer preserved the fusion protein in a non-aggregated state but was harmful to cells. Therefore, prior to use, an aliquot of the TAT-mPiC fusion protein was thawed and dialyzed against 3% glycerol/PBS. The purification steps are summarized in Figure 1E. This multi-step purification protocol produces as the final product, a relatively purified fusion protein (only one additional non-specific band was detected on the SDS gels) that includes only the TAT-mPiC sequence (M_w_ ≈ 42 kDa) without any added tags.

### 2.3. Internalization, Localization into the mIM and TAT-mPiC Fusion Protein Processing

**Figure 2:**
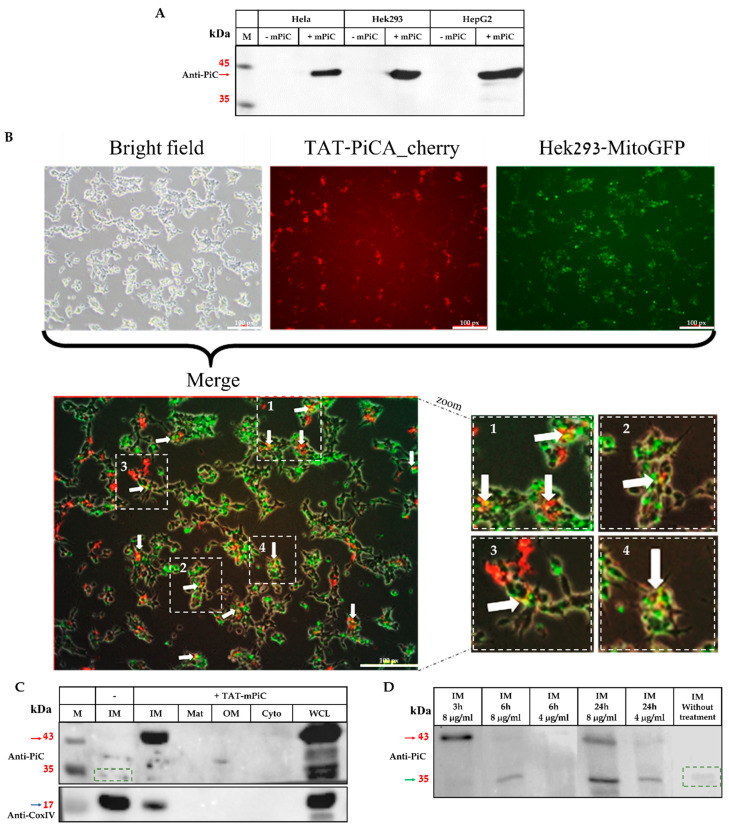
TAT-mPiC fusion protein internalization and processing. (**A**) Three cell lines (HeLa, HEK293, and HepG2) were incubated with the TAT-mPiC fusion protein for 2.5 h, whole cell lysates from cells with or without the addition of the fusion protein were analyzed by Western blot. (**B**) Hek293-MitoGFP cells were incubated with TAT-PiCA-cherry fusion protein (20 µg/mL) for 3 h and a fluorescence microscope was used to obtain images. White arrows indicate yellow spots where MitoGFP and TAT-PiCA-cherry overlap. The white boxes indicate the region of interest, which is enlarged in the inset to highlight colocalization. (**C**) HeLa cells were incubated for 3 h with the fusion protein then the mitochondria were isolated and sub-fractionated and analyzed by Western blot. (**D**) Isolated mitochondrial inner membranes of treated HeLa cells were incubated for different time periods and protein treatment doses and analyzed by Western blot. IM—mitochondrial inner membrane, Mat—mitochondrial matrix, OM—mitochondrial outer membrane, Cyto—cytoplasm, WCE—Whole cell lysate. Red arrow—42 kDa full length fusion protein, green dashed rectangle—endogenous mPiC protein, green arrow—the 34 kDa processed exogenous mPiC protein.

To examine the ability of TAT to deliver the human PiC into cells, HeLa, HEK293, and HepG2 cells were incubated with the TAT-mPiC fusion protein. Cells with or without treatment were lysed and analyzed by Western blot using anti-PiC antibodies. As shown in Figure 2, the TAT-mPiC fusion protein (42 kDa) entered the treated cells and was detectable as early as 2.5 h post treatment (Figure 2A).

For further examination, Hek293-mitoGFP cells were incubated with TAT-PiCA-cherry. The red-fluorescent fusion protein entered cells, and overlayed with the green mitochondria, as can be seen by the yellow staining (Figure 2B). Next, we examined whether the exogenously delivered mPiC, in the form of TAT-mPiC fusion protein, could be delivered to its natural location, the mIM, and processed within the mitochondria. HeLa cells were sub-fractionated (see Methods) and analyzed by Western blot using anti-PiC antibodies. As shown in Figure 2C, by three hours after the start of the incubation, the TAT-mPiC fusion protein (42 kDa) had entered the cells, reached the mitochondria, and localized within the mIM. TAT-MTS cleavage by mitochondrial peptidases likely began within six hours, leaving the mPiC protein (M_w_ ≈ 34 kDa) within the mIM and was dose- and time-dependent, as can be seen in Figure 2D and Figure S2.

### 2.4. PiC-siRNA Reduces the Proliferation of HeLa Cells, While Treatment with TAT-mPiC Fusion Protein Rescues Their Proliferation Ability

**Figure 3:**
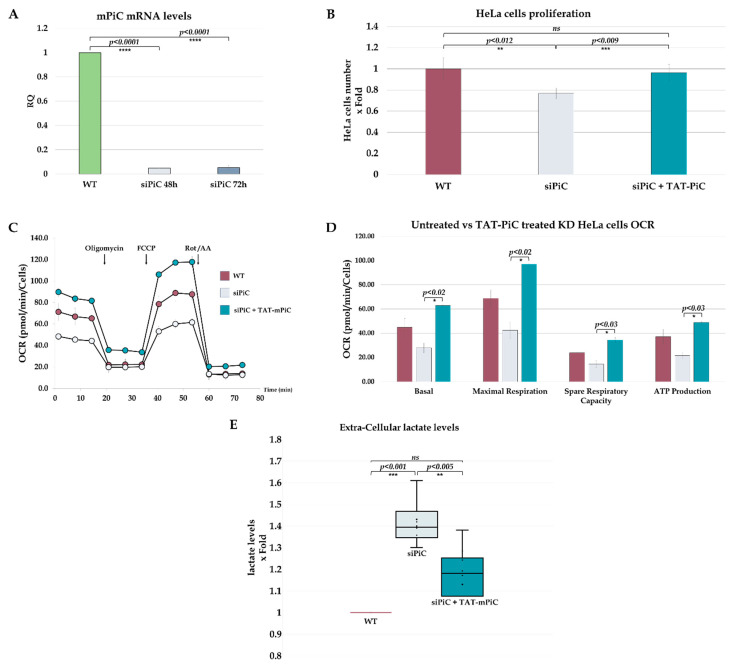
The TAT-mPiC effect on the cells’ energetic profile. (**A**) Relative quantification (RQ) of mPiC mRNA levels by RT-PCR analysis of HeLa cells exposed to siPiC for 48 and 72 h. HeLa cells were incubated with siPiC for 48 h, 6 µg/mL of TAT-mPiC was added for another 24 h. (n = 3) (**B**) The cells’ number was evaluated by trypan blue staining after 72 h (n = 6). (**C**) OCR of WT and siPiC HeLa cells with or without treatment was measured using Seahorse. For statistical purposes, eight independent experiments were performed with at least 4 replicates each. Values are shown as the mean of pmolO2/minute/cell. (**D**) Quantification of two independent experiments with at least 4 replicates each. All folds were compared to WT cells. (**E**) The extra-cellular lactate levels were measured, all results were related to WT (=1), each circle represents an individual measurement. The line represents the median, and X represents the average (n = 8). Results are presented as mean ± STD. For statistical analysis, a *t* test was used (ns *p* > 0.05; * *p* ≤ 0.05; ** *p* ≤ 0.01; *** *p* ≤ 0.001; **** *p* ≤ 0.0001).

Since it is difficult to procure patients’ cells and knockout of the essential endogenous mPiC is not feasible, we knocked down mPiC by siRNA transfection (siPiC) [6]. The siRNA sequence targets cDNA of exons 6,7, and 8, resulting in silencing of both PiC isoforms. siPiC decreased mRNA-PiC levels by 95% and 93% at 48 and 72 h post-transfection, respectively (Figure 3A). Knockdown of endogenous mPiC reduced HeLa cell growth in a dose-dependent manner (Figure S3A,B); however, mPiC-KD HeLa cells treated with the TAT-mPiC fusion protein regained the ability to grow at a rate similar to that of untreated cells. Proliferation of siPiC transfected cells was reduced to 0.77 ± 0.05-fold compared to non-manipulated cells (*p* < 0.012). However, proliferation of siPiC transfected cells treated with TAT-mPiC fusion protein was similar to that of non-treated manipulated cells (0.96 ± 0.08) (Figure 3B).

### 2.5. mPiC-KD Cells Exhibit the Expected OXPHOS Defect, Which Can Be Rescued by Treatment with the TAT-mPiC Fusion

Because PiC is the main carrier mechanism for Pi transfer into the matrix, and Pi is required for OXPHOS, we hypothesized that PiC deficient cells would exhibit a defect in OXPHOS, that could be recoverable by treating the cells with TAT-mPiC. As expected, basal OCR decreased to ×0.62 upon siPiC and increased to ×1.40 upon treatment. Maximal Respiration decreased to ×0.61 upon siPiC and increased to ×1.41 upon treatment. Spare respiratory capacity decreased to ×0.61 upon siPiC and increased to ×1.44 upon treatment. PiC deficiency resulted also in a decrease in ATP-linked OCR (i.e., Pi-dependent OCR) to ×0.58 upon siPiC and increased to ×1.32 upon treatment, which was estimated by subtracting the oligomycin-insensitive OCR (i.e., OCR after inhibiting the ATP synthase with oligomycin) from the basal OCR (Figure 3D). Consistent with lower ATP-linked OCR, lactate levels in si-mPiC-treated cells were significantly higher than in control untreated cells (1.4-fold, *p* < 0.001), the addition of TAT-mPiC decreases the extra-cellular lactate levels similar to that of untreated cells (1.11 ± 0.18, *p* < 0.05) (Figure 3E). Also noted in PiC-deficient cells was a decrease in the maximal electron transport chain activity (FCCP-driven OCR), which can be interpreted as secondary to PiC loss. Treatment of PiC-depleted cells with TAT-mPiC restored ATP-linked OCR, lowered lactate levels and also increased the electron transport chain capacity (Figure 3C,D). These results suggest that treatment with TAT-mPiC fusion protein significantly restores OXPHOS in mPiC-KD cells and, along with that, normalizes glycolysis and OXPHOS capacity. Measuring ATP levels by an independent approach demonstrated that the mitochondrial derived ATP level was lowered to 0.81-fold upon mPiC-KD and increased by 1.12-fold when treated with TAT-mPiC protein compared to untreated si-mPiC KD cells (Figure S4B).

It should be emphasized that eight independent experiments were conducted, revealing a consistent trend in all of them, strongly suggesting that treatment with TAT-mPiC fusion protein restores the bioenergetic profile of cells with dysregulated mitochondrial Pi transport.

### 2.6. TAT-mPiC Fusion Protein Carries Pi and Cu to the Mitochondrial Matrix

To test the biological function of the TAT-mPiC fusion protein as a mitochondrial carrier for both Pi and Cu, upon its translocation within the mIM of treated cells, we isolated mitochondria from HeLa cells and measured P_i_ and Cu levels in mitochondrial lysates of control non-manipulated cells, si-mPiC KD cells and si-mPiC KD cells treated with TAT-mPiC fusion protein.

**Figure 4:**
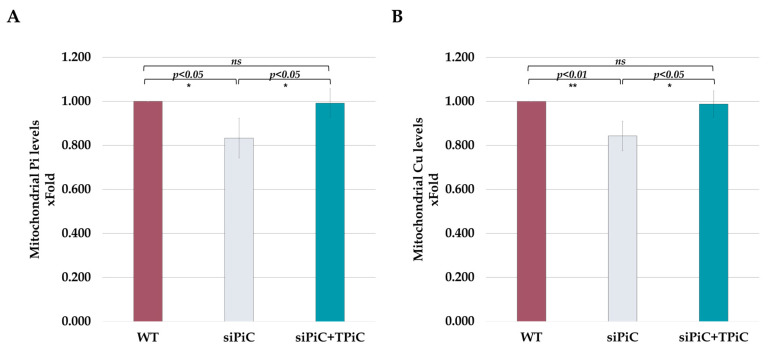
Treatment with TAT-mPiC fusion protein replenishes P_i_ and Cu levels within the mitochondria. After 48 h of incubation with siPiC, cells were incubated with the TAT-mPiC fusion protein (4 µg/mL) for another 24 h. Cells were lysed, the mitochondria were isolated, the mitochondrial P_i_ and Cu levels were measured using commercial kits (see Methods). (**A**) Mitochondrial inorganic phosphate levels (n = 7). (**B**) Mitochondrial copper levels (n = 4). Results are presented as mean ± STD. For statistical analysis, a *t* test was used (ns *p* > 0.05; * *p* ≤ 0.05; ** *p* ≤ 0.01).

Mitochondrial inorganic phosphate levels were significantly decreased to 0.83 ± 0.09-fold upon mPiC knockdown. However, these levels were restored in mPiC-KD cells treated with TAT-mPiC (4 µg/mL) and increased to 0.99 ± 0.06-fold of control cells (Figure 4A).

Mitochondrial Cu levels were significantly reduced to 0.84 ± 0.06-fold upon mPiC knockdown. These levels were restored in mPiC KD cells treated with TAT-mPiC fusion protein (4 µg/mL) and increased to 0.98 ± 0.06-fold of control cells (Figure 4B). These results further suggest that TAT-mPiC fusion protein successfully integrates into the mIM, and its carrier activity is restored upon TAT-mPiC fusion protein treatment.

### 2.7. TAT-mPiC Fusion Protein Restores the Mitochondrial Activity of Cells Harboring Various Mitochondrial Defects

**Figure 5:**
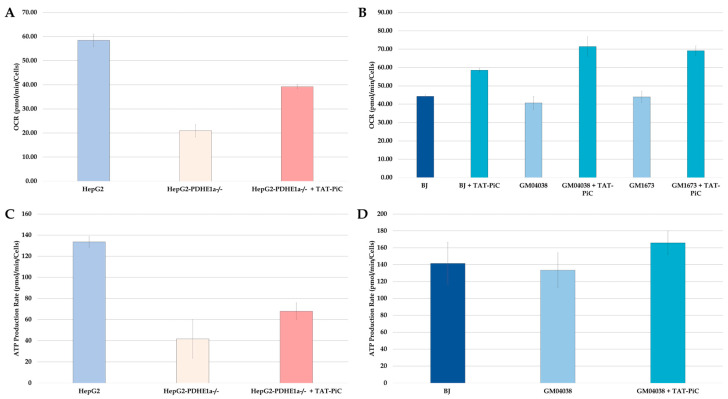
TAT-mPiC fusion protein effect on the basal oxygen consumption rate of cells mutated or lacking other mitochondrial proteins, after 24 h incubation with the fusion protein. Basal OCR of WT HepG2 and HepG2-PDHE1a^−/−^ (Clone5) (n = 3) (**A**), or normal fibroblasts (BJ), fibroblasts from Friedreich Ataxia patient (GM04078 cells), fibroblasts from MMA patient (GM01673 cells) with or without treatment (n = 3) (**B**) were measured using the Seahorse system. Total ATP Production Rate of WT HepG2 and HepG2-PDHE1a^−/−^ (n = 3) (**C**), Normal fibroblasts (BJ) and fibroblasts from Friedreich Ataxia patient (GM04078 cells) (n = 3) (**D**) with or without treatment was measured using the Seahorse system. Results are presented as mean ± STD.

TAT-mPiC fusion protein was added to cells with mitochondrial defects other than MPCD, to examine its possible beneficial effect on these cells’ energy profile. PDH-E1α^−/−^ (Pyruvate dehydrogenase E1 subunit CRISPR knockout) HepG2 cells, which lack the E1α subunit of the pyruvate dehydrogenase complex, as well as GM04078 and GM01673 cells derived respectively from Friedreich Ataxia (FRDA) and Methylmalonic Aciduria (MMA) patients, were treated with the TAT-mPiC fusion protein. Our results show that the basal OCR of HepG2/E1α^−/−^ cells was reduced to 0.35-fold compared to unmanipulated HepG2 cells and increased by 1.87-fold upon treatment with TAT-mPiC fusion protein compared to HepG2/E1α^−/−^, untreated cells (Figure 5A). The basal OCR of BJ normal fibroblasts, GM04078 and GM01673 cells increased to 1.32-, 1.75-, and 1.57-fold, respectively, upon treatment (Figure 5B).

The total ATP levels in HepG2/E1α^−/−^ cells increased to 1.62-fold, and in GM04078 cells to 1.24-fold when treated with TAT-mPiC protein compared to untreated cells (Figure 5C,D). These findings suggest that treatment with TAT-mPiC fusion protein of mutated cells or those lacking mitochondrial proteins other than mPiC, apparently increases Pi levels within the mitochondrial matrix, leading to more ATP production and finally resulting in improved bioenergetic profiles of these cells. However, these preliminary results should be further studied in follow-up experiments.

## 3. Discussion

Primary mitochondrial diseases are among the most frequent and severe inherited metabolic disorders, affecting approximately 1 in every 4300 live births [10]. These diseases result in impaired OXPHOS or other disruptions to mitochondrial structure and function, including abnormalities in mitochondrial ultrastructure [10]. They exhibit marked genetic, mechanistic, and clinical heterogeneity. Mutations of almost 400 genes across two genomes have been linked to primary mitochondrial diseases. Patients may exhibit symptoms in almost any tissue and organ in the body, leading to significant diagnostic challenges. This complexity contributes to a lack of therapeutic options for patients and nearly all mitochondrial disorders currently lack curative therapies. Management for most patients is primarily supportive. However, in the last decade, due to major advances in defining the causes and mechanisms of these diverse disorders, new therapies are being developed in the laboratory and are entering human clinical trials. One such approach is TAT-mediated E/PRT; however, this approach has mainly been limited to replacement of soluble mitochondrial proteins.

In this study, we designed, produced, and purified a TAT-mPiC fusion protein for the replacement of mPiC. The selection of an appropriate protein transduction peptide (PTD) was a critical step. According to modern concepts, there are several mechanisms of PTD penetration into the cells that can be broadly divided into the energy-independent direct penetration and energy-dependent pathways (various types of endocytosis) [23]. Our aim was to circumvent the endo-lysosomal route. According to several reviews on PTDs [11,23,24], the TAT peptide has been shown to preferentially translocate directly into the cytosolic compartment. This suggests that our TAT-fusion protein is able to reach the cytosol without being sequestered within lysosomes, thereby avoiding lysosomal degradation of the fusion protein. This is one of the intrinsic advantages of TAT-fusion proteins such our TAT-mPiCA fusion protein”.

**Figure 6:**
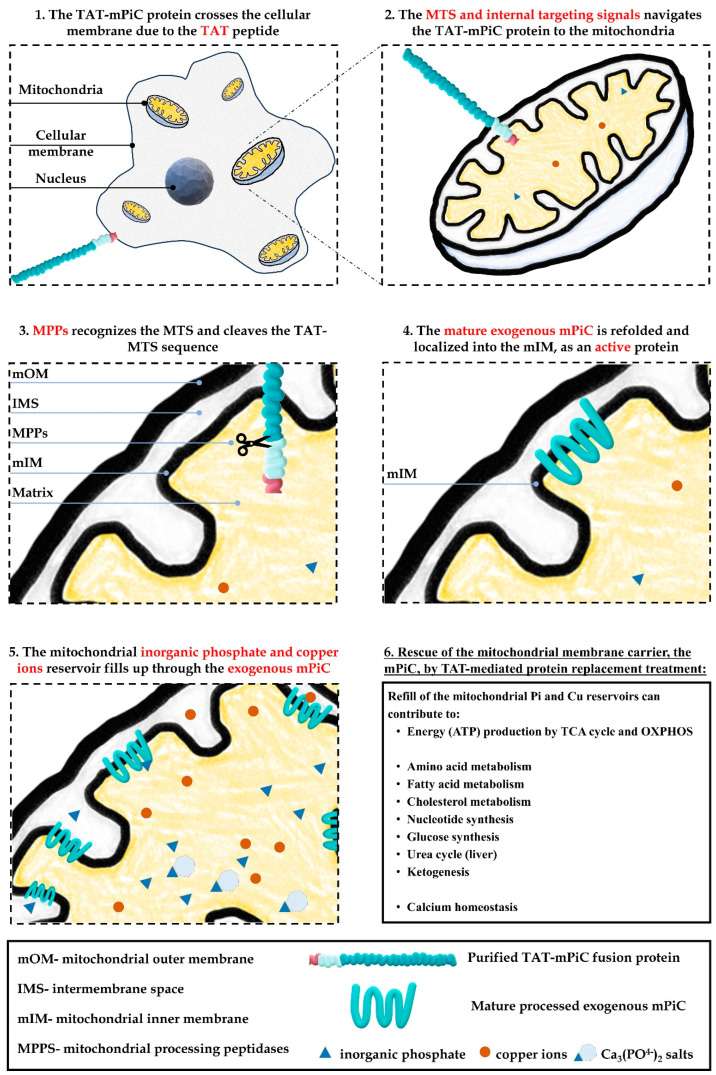
Schematic representation of TAT-mPiC fusion protein mechanism of action and its possible contribution to mitochondrial activities in cells harboring mPiC deficiency or other mitochondrial defects.

The fusion protein was delivered into the mitochondria and localized within the mIM, mPiC’s cellular location, and as a processed protein (Figure 6). Treatment of mPiC-KD cells with the TAT-mPiC fusion protein restored cell growth and bioenergetic capabilities, as measured by OCR and lactate secretion. Most importantly, treatment with TAT-mPiC fusion protein restored delivery of Pi and Cu into the mitochondria of deficient cells. Thus, TAT-mPiC fusion protein may potentially treat some of the foremost MPCD symptoms, such as hypotonia and exercise intolerance, caused mainly by a low ATP generating capacity and lactic acidosis due to impaired oxidative phosphorylation.

The purification process of the TAT-mPiC fusion protein was challenging since it is a highly hydrophobic membrane- carrier and required calibrations at every step, pre- and post-expression of the fusion protein. The transformation and induction were calibrated in seven different hosts with three different IPTG concentrations, several bacterial growth temperatures and durations were assessed to achieve an adequate production level (our unpublished results). Then, a multiple-step purification protocol was necessary, using an affinity column to bind the protein, followed by an additional step of cleaving the His-TEV_rec_ tag using a His-tag TEV enzyme, and then running on a second affinity column; all aimed at binding as much of the non-specific proteins as possible, including the cleaved His-TEV_rec_ and the His-tagged TEV enzyme, leaving the desired TAT-fusion protein in the flow-through of the second affinity column. The purification protocol resulted in a relatively purified TAT-mPiC fusion protein, without any unrequired tags or other proteins.

Even though the fusion protein was initially denatured in 6 M urea buffer, passed through different buffers and finally used in a 3% glycerol/PBS buffer, we assumed that the protein was in an intermediate 3D confirmation. However, the natural intra-cellular refolding mechanism of nuclear-encoded mitochondrial proteins meant that it was not necessary to fully refold it (and probably impossible). During their import process, mIM proteins are synthesized in the cytosol, navigate to the mitochondria according to the MTS and must be unfolded, passing through the membrane in a single strand, by the mitochondrial Tim-Tom machinery. The MTS is the first to enter and reach the matrix, and it is cleaved by the mitochondrial processing peptidase (MPP), and the mature mPiC is simultaneously embedded into the mIM by the TIM22 and internal targeting signals [25].

The TAT sequence’s ability to cross membranes is bi-directional, so the fused cargo moves both in and out of the mitochondria. This means that an intra-mitochondrial processing step to cleave the MTS was essential to remove the TAT sequence to guarantee that the mPiC will be trapped inside the mitochondria. Our results show that the exogenous mPiC, in the form of TAT-fusion protein, entered cells and localized into the mIM. Once delivered, the TAT-MTS sequence was cleaved by the mitochondrial proteases and the mature mPiC was effectively embedded and integrated into the mIM, in the same manner as an endogenous protein. In addition, the imported mPiC protein needs to successfully refold into its correct 3D structure, to guarantee the appropriate carrier activity. This was evidenced by the functional assays that demonstrated improved cellular energy metabolism, and enhanced phosphate and copper transfer, confirming the biological efficacy of the delivered mPiC in the form of a fusion protein.

A range of serious diseases is known to be caused by deficiencies in mitochondrial carriers. According to our results, this purification protocol can be applied to produce and purify a variety of mitochondrial membrane carriers as TAT-fusion proteins, for treatment of such deficiencies as those of mitochondrial ATP/ADP translocase (ANT), carnitine-acylcarnitine translocase (CACT), and dicarboxylate carrier (DIC), using the functional human protein to replace the mutated one.

Moreover, since the purification process of hydrophobic proteins is difficult and challenging, it is worth trying to use this purification protocol and the TAT-mediated strategy to find treatment for other disorders characterized by membranal-protein deficiencies. These include deficiencies in the cellular membrane, for example cystic fibroses transmembrane conductance regulator (CFTR) [26], and in other cellular organelle membranes, for example Lamin A deficiency in the nucleus envelope seen in Progeria (Hutchinson-Gilford Progeria Syndrome) [27], and SEC61 translocon deficiency [28] in the ER membrane.

Serving also as a carrier for Cu, mPiC deficiency leads also to less Cu import. This can lead to several significant issues since mitochondria contain critical cuproenzymes, including cytochrome c oxidase and superoxide dismutase. Insufficient Cu levels can lead to reduced COX metalation and assembly and less superoxide dismutase SOD1 activity [7]. Collectively, P_i_ and Cu are critical for mitochondrial health and function. mPiC lack leads to a lowered bioenergetic profile in MPCD patients. Indeed, as demonstrated in this study, in TAT-mPiC fusion protein treated cells, the OCR and ATP production rate were restored to the levels seen in control cells, and mitochondria regained their ability to actively deliver P_i_ and Cu to the mitochondrial matrix (Figure 4A,B).

Taking into consideration the main role of mitochondria in cells’ energy metabolism, mitochondrial pH balance maintenance, and the numerous metabolic pathways performed by mitochondria (such as amino acid metabolism, fatty acid metabolism, cholesterol metabolism, nucleotide synthesis, glucose synthesis, urea cycle (liver), ketogenesis, calcium homeostasis, etc. Figure 6, panel 6), it is worth considering conducting a screen to test whether adding mPiC, in the form of TAT-mPiC, to cells suffering from mitochondrial deficiencies other than MPCD, can moderately increase mitochondrial P_i_ and Cu levels and consequently benefit these cells. Our results show that the carrier’s activity can affect the energy profile of cells of patients that have deficiencies in PDHE1a, FXN, and MMA (Figure 5A–D). This suggests that more P_i_ and Cu inside the mitochondrial-matrix contribute to many other mitochondrial activities (Figure 6) requiring mainly Pi (but also Cu), and thus may be curative for the down-stream defects caused by various gene deficiencies. This finding is remarkable and could suggest the TAT-mPiC fusion protein as a potential treatment combined with other supplements and drugs to treat various diseases characterized by low mitochondrial ATP production. However, these preliminary results should be further evaluated in follow-up studies.

This study was conducted in vitro, however, in vivo testing in animal models is crucial to assess the systemic effects and potential therapeutic benefits of TAT-mPiC treatment in MPCD or other mitochondrial disorders.

As already stated, the current clinical management of mitochondrial disorders remains predominantly supportive, focusing on symptomatic treatment. Although vitamins and cofactors are regularly recommended for patients with primary mitochondrial diseases, sometimes as a ‘cocktail’, there is no proven efficacy for these agents outside the disorders of vitamin/cofactor metabolism and transport [10].

Yet, in the past few years, new treatment strategies have been proposed for mitochondrial disorders. These approaches act on different disease mechanisms and can be divided into strategies acting on common pathways, thus relevant to different mitochondrial diseases, and specific disease-oriented strategies [29]. One group includes strategies aimed at activation of mitochondrial biogenesis, regulation of mitochondrial dynamics and mitophagy, bypass of OXPHOS defects, mitochondrial replacement therapy, and chronic hypoxia. Another group involves scavenging of specific toxic compounds, supplement of nucleosides and nucleotides, cell replacement therapies, gene therapy, E/PRT, shifting mutant mtDNA heteroplasmy, and stabilizing mutant tRNAs. Some of these strategies have been proven effective only in preclinical models while others have already been successfully applied to patients with mitochondrial diseases [29].

The CPP-mediated E/PRT approach, as represented in the current study, is in its initial stages, with Nomlabofusp (previously known as CTI-1601) being the most developed drug. Nomlabofusp is a CPP-fusion protein designed to deliver human frataxin protein to mitochondria. In March 2024, Larimar announced the first dosing of an individual with FRDA in a long-term open label extension study comprising daily injections of Nomlabofusp [30], supporting promise for this therapy approach.

The approach of TAT-fusion proteins shows great promise for emerging novel therapies, mainly for mitochondrial diseases, as described in this study; however, there are still some challenges and limitations to overcome, such as stabilization of the TAT-fusion proteins, that is necessary to delay of degradation of the molecules by enzymes circulating in the plasma. Another possible limitation is the immune response to administration of the TAT-fusion proteins. TAT sequence, as well as other protein transduction peptides, originate from non-human proteins/peptides and therefore are usually novel to the organism, thus possibly evoking an immune response. Additionally, fusing TAT with a protein will most likely result in the generation of new epitopes that might also elicit an immune response. In addition, in the case of genetic metabolic disorders such as mitochondrial disorders, the replacement of the deficient protein would have to be on a regular, chronic basis, causing additional problems regarding the immune response to the delivered TAT-protein modalities. Although numerous reports have demonstrated that it is possible to safely inject/deliver TAT-fusion protein into animal models [31,32] as well as to humans [30], particularly careful evaluations should be performed to search for non-specific toxicity of TAT-fusion proteins molecules.

MPCD can lead to severe and life-threatening complications early in life, thus, TAT-mediated replenishment of mPiC could be an early treatment strategy in newborns vulnerable to crises when mPiC is deficient. mPiC is the first mitochondrial-membrane **carrier** to be delivered to cells via the TAT-delivering system. The successful delivery of TAT-mPiC and its integration into mitochondrial inner membrane have significant implications for treating mitochondrial disorders that are linked to mPiC and other mitochondrial-membrane proteins’ deficiencies as well. Moreover, our research dealing with the mitochondrial carrier, mPiC, takes the CPP-mediated E/PRT approach one step forward, suggesting its possible application not only for mutated intra-cellular soluble proteins, but rather for various mutated highly hydrophobic membranal proteins of cells that are involved in many human diseases.

Collectively, our results introduce the multi-step protocol as a strategy for membrane/hydrophobic protein purification and propose the TAT-mPiC fusion protein as a potential early treatment strategy for newborns with MPCD, or as a combination for treating other mitochondrial diseases.

## 4. Materials and Methods

### 4.1. Construction of the Plasmids Encoding TAT-mPiCA Fusion Proteins

A plasmid encoding the His-TAT-MTScs-FXN fusion protein, pre-existing in our laboratory [16], was cut with BamH1 and XhoI restriction enzymes to remove the MTScs-FXN coding sequence, thereby obtaining the cut vector fragment. The full length human mPiC (isoform A), including its MTS (custom peptide sequence purchased from Syntezza Bioscience Ltd., Jerusalem, Israel), was amplified by PCR using the following synthetic oligonucleotide primers covering the whole coding sequence:

5′ GACCGGATCCTTCTCGTCCGTGGCGCACCTGGCGCGG 3′ (sense),

5′ AAGAAGAAGCTTGGGTTAACTCAGTAGCTCGAGCCC 3′ (anti-sense).

The PCR product was cut with the same BamHI/XhoI enzymes. This fragment was ligated with the vector fragment using Quick Ligation Kit (New England Biolabs, Ipswich, MA, USA), producing the plasmid pET28-TAT-mPiCA. A plasmid encoding the Vim-mCherry sequence, generously provided by Prof. Yoav Shaul, was amplified by PCR using the following synthetic oligonucleotide primers: 5′ ACGAAGCTTGGGTTAACTCAGGTGAGCAAGGGCGAGGAG 3′ (sense), 5′ ACACCTCGAGGGTACCGGTTACTTGTACAGCTC 3′ (anti-sense), producing a mCherry fragment containing the last 20 base pairs of the PiCA sequence( without the stop codon(, with HindIII site at the 5′ and a XhoI site at the 3′. The His-TAT-PiCA plasmid and mCherry fragment were cut with HindIII and XhoI restriction enzymes, the mCherry fragment was cloned downstream of the His-TAT-PiCA sequence using Quick Ligation Kit (New England Biolabs, Ipswich, MA, USA), producing the plasmid encoding the His-TAT-PiCA-mCherry fusion proteins schematically presented in Figure S5.

Next, the full length human mPiC isoform A, including the TAT sequence at the N’ terminus (TAT-mPiCA fragment) was amplified, using the pET28-TAT-mPiCA as a template, by PCR using the following synthetic oligonucleotide primers:

5′ TATGGAGAATCTTTACTTTCAGGGGAGGAAGAAGCGGAGACAGCG 3′(sense),

5′ GGCTTTGTTAGCAGCCGGATCCTCGAGCTACTGAGTTAACCCAAGC 3′ (anti-sense).

To create an open pET15b plasmid, the plasmid was amplified by PCR with a high fidelity Taq polymerase (Phanta Flash, Biogate, Israel) using the following synthetic oligonucleotide primers:

5′ CTCGAGGATCCGGCTGCTAAC 3′ (sense),

5′ CCCCTGAAAGTAAAGATTCTC 3′ (anti-sense).

The product was inserted into an open pET15b plasmid encoding the 5′ His-TEV (recognition site) 3′ sequence, by Gibson Assembly^®^ (New England Biolabs, Ipswich, MA, USA) according to the manufacturer’s instructions, producing the plasmid pET15b-TAT-mPiCA encoding the sequence for His-TEV_rec_-TAT-mPiCA (Figure S6). All plasmid constructs were confirmed by sequencing analyses.

### 4.2. Protein Expression

pET28-TAT-MTS-PiCA-cherry plasmid was transformed into the BL21-C41(DE3) *Escherichia coli* strain. Protein expression was performed using the methods in [13,14,15,16,17] with modifications described here: Bacteria were grown until an OD_600_ of 0.7–0.8 was reached. Protein expression was induced by incubating with 1 mM isopropyl-1-thio-D-galactopyranoside (IPTG), at 30 °C overnight. The cells were then centrifuged (4000× *g*, 20 min), and the pellet was frozen at −80 °C at least for 30 min. The frozen cells were thawed and suspended in lysis buffer (10 mM Tris-HCl, pH 8.0; 0.1 M NaCl; 0.1 mM NaH_2_PO_4_; 0.2 mg/mL lysozyme; 1 mM phenylsulfonyl fluoride (PMSF)) for 30 min at room temperature, followed by sonication. Cells were then centrifuged at 29,000× *g* for 60 min. The supernatant was removed and the pellet (containing inclusion bodies) was suspended in denaturation buffer (6 M urea; 10 mM Tris-HCl, pH 8.0; 0.1 M NaCl; 0.1 mM NaH_2_PO_4;_ 2 mM βME) and continuously stirred at 4 °C overnight. The solution was cleared by centrifugation at 29,000× *g* for 60 min. The supernatant, containing denatured proteins, was collected and marked as inclusion bodies (IB).

pET15b-TAT-mPiCA plasmid was transformed into Rosetta *Escherichia coli* strain and bacteria were grown until an OD_600_ of 0.7–0.8 was reached. Protein expression was induced by 1 mM IPTG, at 37 °C for 4 h. The cells were then centrifuged (4000× *g*, 20 min), and the pellet was frozen at −80 °C for at least 30 min. The frozen cells were thawed and suspended in lysis buffer [20 mM Tris-HCl, pH 8.0; 0.05 M NaCl; 0.05 mM NaH_2_PO_4_; 0.2 mg/mL lysozyme; 1 mM phenylsulfonyl fluoride (PMSF)] for 30 min at room temperature, followed by sonication. A sample was taken from the lysed cells and marked as whole cell extract (WCE). The remaining cells were then centrifuged at 29,000× *g* for 60 min. The supernatant (marked as soluble fraction; Sol) was removed and the pellet (containing inclusion bodies) was suspended in denaturation buffer (6M urea; 20 mM Tris-HCl, pH 8.0; 0.05 M NaCl; 0.05 mM NaH_2_PO_4;_ 2 mM βME) and continuously stirred at 4 °C overnight. The solution was cleared by centrifugation at 29,000× g for 60 min. The supernatant, containing denatured proteins, was collected, and marked as inclusion bodies (IB).

### 4.3. TAT-mPiCA Fusion Protein Purification

The TAT-fusion proteins’ purification was performed using the method in [33] with modifications described in detail here: The TAT-PiC-cherry denatured fusion protein in 6 M urea (see above) was filtrated and a final concentration of 10 mM imidazole was added. The protein was subjected to immobilized metal affinity chromatography using 5 mL His-Trap columns (GE Healthcare, Chicago, IL, USA) and was eluted with a linear imidazole gradient of 10–500 mM. Fractions containing the fusion protein were pooled. Desalting and buffer exchange to phosphate-buffered saline (PBS) with 5% glycerol was performed using dialysis. The fusion protein was aliquoted and kept at −20 °C.

The His-TEV-TAT-mPiC denatured protein preparation in 6 M urea (see above) was filtrated and imidazole was added to a final concentration of 20 mM. The protein was subjected to immobilized metal affinity chromatography using 5 mL His-Trap columns (GE Healthcare, Chicago, IL, USA) and was eluted with a linear imidazole gradient of 20–500 mM. Fractions containing the protein preparation were pooled.

The pooled fractions were incubated with pre-dialysis buffer (3 M urea; 20 mM Tris-HCl, pH 8.0; 0.05 M NaCl; 0.05 mM NaH_2_PO_4_; 2 mM βME) in a dialysis bag at 4 °C for 3 h, and then the TEV enzyme was added to the bag and continuously stirred at 4 °C overnight, to cleave the protein and lower the high concentration of imidazole.

The resulting solution was subjected to a second affinity chromatography His-Trap column (GE Healthcare, Chicago, IL, USA); collecting this time the flow-through of the column-run. The flow-through fraction was subjected to desalting and buffer exchange into PBS containing 10% glycerol, via dialysis; thus, obtaining the TAT-mPiCA fusion protein. The fusion protein was aliquoted and kept at −20 °C. Prior to use, the fusion protein was thawed and dialyzed against PBS containing 3% glycerol for three hours. The protocol for purifying TAT-mPiCA fusion protein is schematically illustrated in Figure 1E. All purification procedures were carried out using the FPLC system ÄKTA (GE Healthcare, Chicago, IL, USA).

### 4.4. Protein Concentration

Protein concentration was measured according to the Bradford method [34], using Bradford reagent and a standard curve of BSA. Protein concentration was determined at a wavelength of 595 nm.

### 4.5. Separation of Proteins by Gel Electrophoresis

Samples from the various protein sub-cellular fractions (WCE, Sol and IB) and purification steps were separated on 12% SDS-PAGE gels and stained with Coomassie blue for visualization of the proteins.

### 4.6. Western Blot Analysis

Following separation on SDS-PAGE gel, the proteins were electro-transferred onto a polyvinylidene fluoride (PVDF) membrane (Bio-Rad, Hercules, CA, USA), as described in [13,14,15,16,17,33]. The membranes were then blotted with one of the following antibodies: rabbit anti-SLC25A3 (anti-PiC 1:2000; Proteintech Group, Inc., Rosemont, IL, USA, Cat# 15855-1-AP) mouse anti-His (1:5000; A_2_S Technologies, Yavne, Israel, Cat#A00174), mouse anti-COXIV (1:5000; Abcam, Cambridge, UK, Cat#ab33985), mouse anti-E1α (1:10,000; Invitrogen™, Waltham, MA, USA, Cat#A21323). The secondary antibodies used were goat anti-rabbit or goat anti-mouse HRP conjugated (1:10,000) (Jackson ImmunoResearch Laboratories Inc., West Grove, PA, USA). Band visualization was performed using the EZ-ECL chemiluminescence detection kit (Biological Industries, Beit Haemek, Israel).

### 4.7. Cell Lines

HeLa, HEK293, HepG2, and BJ cell lines were obtained from the American Type Culture Collection (ATCC-Manassas, VA, USA). HeLa cervical carcinoma cells (ATCC-CCL-2), HEK293 human embryo kidney cells (ATCC-CRL-1573), HepG2 hepatocellular carcinoma cells (ATCC-HB-8065), BJ normal fibroblasts (ATCC-CRL-2522), and Hek293-MitoGFP (our engineered cells as described in [35]) cells were grown in DMEM medium supplemented with 10% fetal bovine serum (FBS; Merck, Darmstadt, Germany), 2 mM L-glutamine (Sartorius, Göttingen, Germany), 100 units/mL penicillin, 100 µg/mL streptomycin. (Penicillin-Streptomycin Solution 100X; Biowest, Rue de la Caille, Nuaillé, France).

HepG2-PDH-E1α^−/−^ (CRISPR knockout-Clone5; Lorberboum-Galski H., our unpublished data) cells were grown in RPMI medium supplemented with 10% FBS, 2 mM L-glutamine, 100 units/mL penicillin, 100 µg/mL streptomycin.

Fibroblasts from an MMA patient (methylmalonic aciduria due to methylmalonic Coenzyme A mutase deficiency; GM01673 cells) were obtained from Coriell Cell Repositories (Camden, NJ, USA) and grown in the recommended DMEM medium with 20% FBS and the additives as described for the other cell lines.

Fibroblasts from a Friedreich Ataxia patient (GM04078 cells; fibroblast from skin, arm) were obtained from Coriell Cell Repositories (Camden, NJ, USA) and grown in the recommended medium (Eagle’s Minimum Essential Medium with Earle’s salts) supplemented with 10% FBS and the additives as described for the other cell lines.

All cells were maintained in flasks and grown in a highly humidified atmosphere of 5% CO_2_ at 37 °C.

### 4.8. Internalization of the TAT-mPiC Fusion Protein

Hek293-MitoGFP cells (2.5 × 10^6^ ) were seeded in 100 mm plates and treated with the TAT-mPiC-cherry fusion protein (final concentration 20 μg/mL) or 5% glycerol/PBS (same volume as fusion protein treatment) and incubated for 3 h. Fluorescent images were obtained with the fluorescence microscope at 100× magnification, using GFP and RFP filters.

About 10^7^ HeLa, HEK293 or HepG2 cells were seeded in 150 mm plates and treated with the TAT-mPiC fusion protein (final concentration 15 μg/mL) or 3% glycerol/PBS (same volume as fusion protein treatments) and incubated for 24 h. Internalization of the fusion protein into cultured cells was examined by Western blot analysis of treated cell extracts using anti-PiC antibodies.

### 4.9. Subcellular Fractionation of Cultured Cells

HeLa cells (10^7^) were seeded in 150 mm plates and treated with the fusion protein (final concentration 15 μg/mL) or 3% glycerol/PBS (same volume as fusion protein treatments) and incubated for 24 h. Cells were collected by trypsinization, suspended in PBS, centrifuged (500× *g*, 5 min), and washed once with PBS. Mitochondrial isolation procedures were performed at 4 °C or on ice. The cell pellet was resuspended in MTiso-buffer [3 mM HEPES-KOH (pH 7.4); 210 mM mannitol; 70 mM sucrose; 0.2 mM EGTA; Complete Mini EDTA-free protease inhibitor cocktail tablet (Roche; Basel, Switzerland)], and homogenized with a Dounce homogenizer (WHEATON^®^ SAFE-GRIND^®^ Potter-Elvehjem Tissue Grinder cat# 358005 (PTFE pestle, clearance range: 0.1–0.15 mm)). An equal volume of 340 mM sucrose was added to the homogenate and centrifuged (500× *g*, 10 min) to remove nuclei and non-homogenized cells, and the supernatant was collected. The supernatant was then centrifuged (10,000× *g*, 10 min) to isolate mitochondria as a pellet. The isolated mitochondria were resuspended with 1 mL of 1 mg/mL digitonin buffer. The suspension was mixed intensely for 15 min by vortex. Then, an equal volume of MTiso-buffer was added to stop the digitonin extraction. The suspension was centrifuged (10,000× *g*, 10 min) and the supernatant containing solubilized mitochondrial outer membrane (mOM) and inter-membrane space (IMS) proteins was collected. The pellet containing mitoplast (mIM plus matrix) was resuspended in 0.2 mL MTiso-buffer, gently sonicated, and then centrifuged (100,000× *g*, 30 min), separating the mIM fraction as the pellet and matrix fraction in the supernatant [36].

### 4.10. SLC25A3 Gene KnockDown

esiRNA (MISSION^®^esiRNA, purchased from Merck, Darmstadt, Germany, Cat#EHU089191) against *SLC25A3* gene transcripts was utilized for the knockdown of *SLC25A3*. The *SLC25A3* cDNA target sequence is as follows:

CCCGCAGTGAATGTTCAAAGCCAGAGCAGCTGGTTGTAACATTTGTAGCAGGTTACATAGCTGGAGTCTTTTGTGCAATTGTTTCTCACCCTGCTGATTCTGTGGTATCTGTGTTGAATAAAGAAAAAGGTAGCAGTGCTTCTCTGGTCCTCAAGAGACTTGGATTTAAAGGTGTATGGAAGGGACTGTTTGCCCGTATCATCATGATTGGTACCCTGACTGCACTACAGTGGTTTATCTATGACTCCGTGAAGGTCTACTTCAGACTTCCTCGCCCTCCTCCACCCGAGATGCCAGAGTCTCTGAAGAAGAAGCTTGGGTTAACTCAGTAGTTAGATCAAAGCAAATGTGGACTGAATCTGCTTGTTGATCAGTGTTGAAGAAAGTGCAAAAGGAACTT.

Two hundred and forty microliters of reduced serum medium (Opti-MEM™ I, Gibco™, Waltham, MA, USA, Cat# 31-985-070), 3 µL of TransIT-X2 (Mirus, Madison, WI 53719, USA, Cat#MC-MIR-6000), and 3 µL of a 14 µM *SLC25A3* stock solution (20 nM final concentration per well) were mixed and incubated at room temperature for 30 min. In a 6-well plate, 245 µL of the mix was added to each well, and then 2 × 10^5^ cells/1.5 mL were seeded in DMEM medium supplemented with 10% serum (without antibiotics). Twenty four hours later, the medium was replaced with a complete growth medium for another 24 or 48 h.

### 4.11. Real-Time PCR Analysis of PiC mRNA Levels

HeLa cells (10^6^) and HeLa-KD (10^6^) cells after 48 and 72 h incubation with si-mPiC were collected in three different tubes. Total RNA was extracted from cells using a commercial kit (GENEzol™ TriRNA Pure Kit, New Taipei City, Taiwan, Cat#GZXD200). RNA concentrations were determined using a NanoDrop spectrophotometer (Thermo Scientific, Waltham, MA, USA). One µg of each RNA sample was reverse transcribed using a reverse transcription kit (qScript cDNA Synthesis Kit, Quantabio, Beverly, MA, USA, Cat# 95047-100-2). The resulting cDNA was diluted in DNase-free water (1:10) before quantification mRNA-PiC levels by real-time quantitative PCR. Each sample contained 2 µL cDNA, 1 µL primers, 5 µL SYBR^®^ Green, and 3 µL H_2_O in a total volume of 10 µL per sample. The primers used are listed in Table 1. The data were analyzed using the StepOne™ Software v2.3 (Applied Biosystems, Foster City, CA, USA). All data are expressed as the ratio between the expression level of the target gene mRNA and that of actin.

### 4.12. Proliferation Assays

Forty eight hours after si-mPiC transfection (see above), the medium was replaced with a glucose-free (Gibco™, Thermo Fisher Scientific Inc., Waltham, MA, USA, Cat#11966025) growth medium for 3 h and then the TAT-mPiC fusion protein was added, incubated for another 24 h, and the cell number was evaluated by trypan blue staining (Merck, Darmstadt, Germany, Cat# 93595) using hematocytometer.

### 4.13. Oxygen Consumption and ATP Production Rates

#### 4.13.1. Measuring OCR

Forty eight hours post si-mPiC transfection (see above), medium was replaced with glucose-free medium (Gibco, Thermo Fisher Scientific Inc., USA) for three hours, treatment added, and the plate was incubated for 24 h at 37 °C in a humidified atmosphere of 5% CO_2_. On the day of the experiment, cells were trypsinized with Recombinant Trypsin EDTA solution (Cat#03-079-1B, Sartorius, Göttingen, Germany) and resuspended with warm, freshly prepared glucose-free XF DMEM, pH 7.4 (Agilent Technologies™, Santa Clara, CA, USA, Cat#10-357-5100) containing 1 mM pyruvate and 2 mM glutamine. About 3 × 10^4^ cells/180 μL/well were seeded in Agilent Seahorse XF96 microplates (Cat#NC2151382) pre-incubated with poly-D-lysine. The plate was placed at 37 °C in a humidified atmosphere of 5% CO_2_ for 1 h and then moved to a non-CO_2_ incubator for 1 h. Seahorse XF analysis was performed with Seahorse XFe96 Analyzer (Agilent Technologies Inc., USA). The Seahorse XF Cell Mito Stress Test assay was performed at 37 °C according to the manufacturer’s instructions. Briefly, 18 µM oligomycin (Cat No. 11342; Cayman Chemical, Ann Arbor, MI, USA), 20 µM trifluoromethoxy carbonyl cyanide phenylhydrazone (FCCP) (Cat No. 15218; Cayman Chemical), 10 µM rotenone/antimycin A (Cat No. 13995; Cayman Chemical Santa Cruz Biotechnology, respectively), and 10 µg/mL Hoechst were loaded at the cartridge ports, and the sensor cartridge was calibrated for 15 min. The plate was loaded starting with basal OCR measurement. Then, 20 µL oligomycin was injected to each well (final concentration 1.8 μM), followed by injection of 22 µL FCCP to each well (final concentration 2 μM) and finally 25 µL rotenone/antimycin/Hoechst was injected to each well (final concentration 1 μM for rotenone/antimycin A and 1 µg/mL for Hoechst). The duration of each cycle was 18 min (3 cycles of 3 min mixing and 3 min measurement). Cell numbers were evaluated using a Cytation 5 reader (BioTek^®^, Winooski, VT, USA), and Gen5 Image Prime 3.14 software. Calculations for OCR were done by Wave software (v.2.6.1.53, Agilent Technologies™, Santa Clara, USA).

#### 4.13.2. Measuring ATP Production Rate

The same protocol of the Seahorse XF Cell Mito Stress Test assay was performed but without FCCP injection. The first injection was 20 μL of 18 μM oligomycin A (final concentration 1.8 μM). The second injection was 22 μL of 10 μM rotenone/antimycin A (final concentration 1 μM). The third injection was Hoechst (final concentration 1 µg/mL).

### 4.14. Measuring Lactate Levels

Th growth media of treated and untreated cells were collected, and lactate levels were measured using the ABL90 FLEX blood gas analyzer (Radiometer Medical ApS, Brønshøj, Denmark). Lactate levels were normalized to cell number.

### 4.15. Measuring Phosphate and Copper Levels

Subcellular fractionation was performed using the Abcam protocol [37]. P_i_ and Cu levels were measured within isolated mitochondria using the Abcam ab65622 Phosphate Assay Kit [38] and the Abcam ab272528 Copper Assay Kit [39], respectively, according to the manufacturer’s instructions. Results were normalized to protein concentration, measured using Bradford reagent.

For all experiments measuring lactate, Pi, and Cu levels, HeLa cells were incubated with si-mPiC for 48 h, and then 4 µg/mL of the TAT-mPiC fusion protein or 3% glycerol/PBS (dialysis buffer) was added for another 24 h.

### 4.16. Statistical Analyses

Eight independent Seahorse experiments were conducted; the quantification results are the average of 3 independent biological experiments, each included at least 5 replicates of each sample.

Statistical analysis was performed using Microsoft Excel 365 and an unpaired, two-tailed *t* test was used to determine *p* values.

## Figures and Tables

**Table 1:** Primers used for real-time PCR.

Primer Name	Sense/Anti-Sense	Primer Sequence
SLC25A3 (exons 6–7 and 8)	sense	AGCAGGTTACATAGCTGGAGTC
	anti-sense	TGATACGGGCAAACAGTCCC
β-Actin	sense	CCAACCGCG AGAAGATGA
	anti-sense	CCAGAGGCGTACAGGGATAG

## Data Availability

Data are contained within the article and Supplementary Materials.

## Supplementary Materials

The following supporting information can be downloaded at: https://www.mdpi.com/article/10.3390/ijms26094379/s1.
