# Supplementary material for: Rescue of the First Mitochondrial Membrane Carrier, the mPiC, by TAT-Mediated Protein Replacement Treatment
Source: Int J Mol Sci. 2025 May 5;26(9):4379. doi: 10.3390/ijms26094379 (PMC12073058; doi:10.3390/ijms26094379)
Supplement: Supplementary file 1 [file ijms-26-04379-s001.zip › ijms-3577863-supplementary.pdf]

Rescue of the First Mitochondrial Membrane Carrier, the mPiC, by TAT-Mediated Protein Replacement Treatment

Samar Zabit [1], Orly Melloul [1], Michal Lichtenstein [1], Erin L. Seifert [2] and Haya Lorberboum-Galski [1,*]

[1] Department of Biochemistry and Molecular Biology, The Institute for Medical Research Israel-Canada
(IMRIC), Faculty of Medicine, The Hebrew University of Jerusalem, Jerusalem 9190501, Israel;
samar.zabit@mail.huji.ac.il (S.Z.); orly.elbaz@mail.huji.ac.il (O.M.); michallic@ekmd.huji.ac.il (M.L.)
[2] MitoCare Center, Department of Pathology and Genomic Medicine, Thomas Jefferson University,
Philadelphia, PA 19107, USA; erin.seifert@jefferson.edu
* Correspondence: hayag@ekmd.huji.ac.il

Supplementary Material

Figure S1. Calibrating the TAT-mPiC fusion protein expression and purification processes.

TAT-mPiC fusion protein was expressed in five different hosts (BL21, C41, Rosetta, C43 and HMS) with three different IPTG concentrations (0.25, 0.5 and 1 mM) at 37°C for four hours. WCE from each sample was prepared and separated on 12% SDS-PAGE and Coomassie blue staining (A) and Western blot analysis with anti-PiC antibody 1:5000 was performed (B). (C) Following expression in Rosetta strain E. coli, the fusion protein was purified using two affinity columns, peak samples from the elution of the fusion protein from the first affinity column were pooled, cleaved using TEV enzyme, dialyzed and subjected to a second nickel column. Flow through and peak samples (after imidazole gradient) from the second affinity column elution of the fusion protein were separated on 12% SDS-PAGE and analyzed using Coomassie blue and western blot analysis with anti-PiC antibody (1:1000) and anti-His antibody (1:5000). Uninduced (un), Whole cell extract (WCE), Soluble (Sol), Inclusion bodies (IB); Protein markers on gels are marked by M.

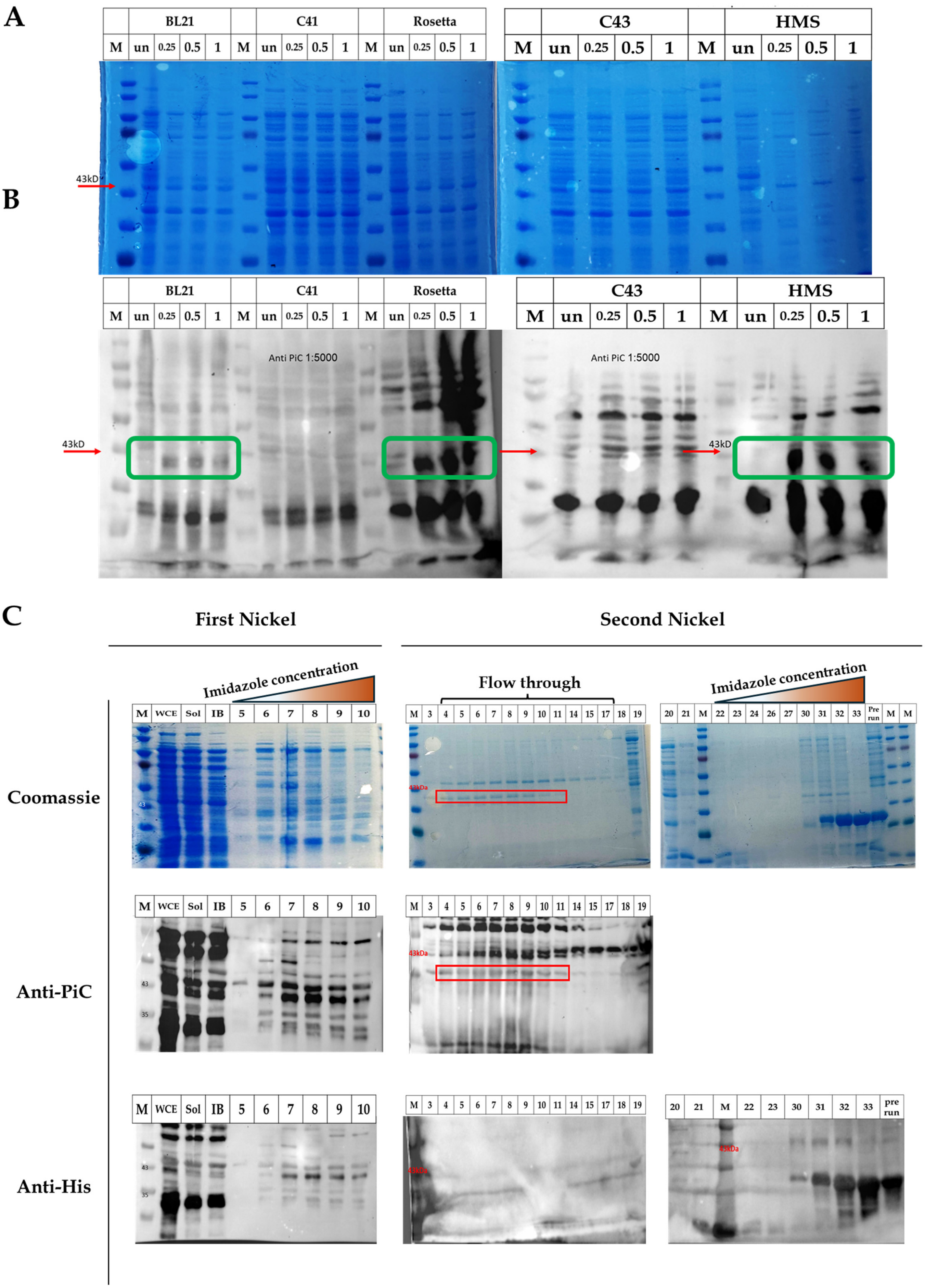
A
B
BL21
C41
Rosetta
C43
HMS
M
un
0.25
0.5
1
43kD
Anti PiC 1:5000
C
First Nickel
Second Nickel
Imidazole concentration
Flow through
Coomassie
Anti-PiC
Anti-His
WCE
Sol
IB
Pre run
43kDa
43
35

Figure S2. TAT-mPiC fusion protein internalization and processing.

Western blot analysis of whole mitochondrial lysates extracted from WT or siPiC HeLa cells incubated with siPiC for 48 hours, then, 0- 8- or 20µg/ml of TAT-mPiC was added for another 24 hours. Lysates were separated on 12% SDS-PAGE and analyzed using western blot analysis with anti-PiC antibody (1:1000), anti-His antibody (1:5000) and anti-E1α antibody (1:10000). Green arrow -unprocessed exogenous mPiC (43 kDa), White arrow processed exogenous mPiC (35 kDa), Red arrow- endogenous mPiC (35kDa). Protein markers on gels are marked by M.

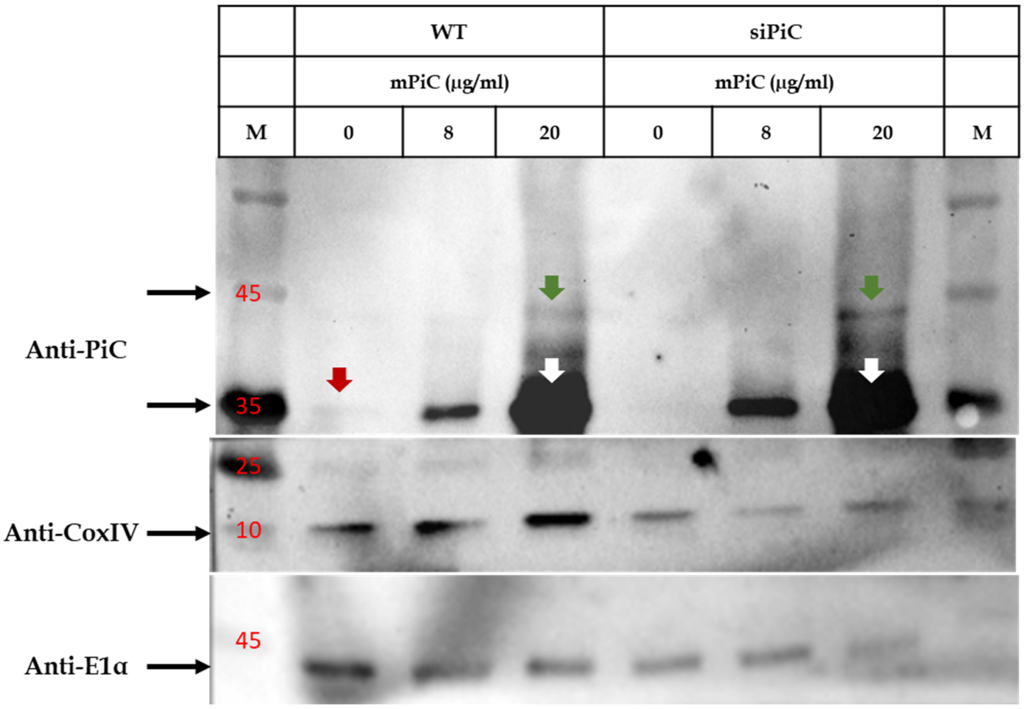

Figure S3. The si-mPiC effect on PiC mRNA levels and cell proliferation is dose dependent. HeLa cells were exposed to different doses of siPiC for 48 hours. (A) Relative quantification (RQ) of mPiC mRNA levels by RT-PCR analysis. In cells exposed to lower concentrations of siPiC (6.6 nM), the PiC mRNA levels were higher compared to PiC mRNA levels in cells exposed to higher concentration (20 nM) (n=3). (B) The cell number was evaluated by hematocytometer; the more siPiC was added to cells, the less cells proliferated (n=3). Results are presented as mean ± STD.

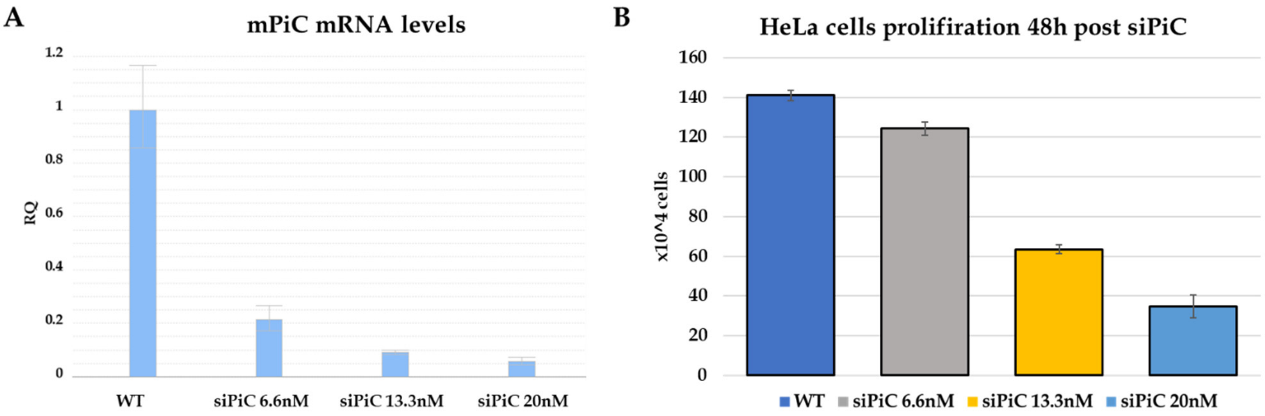

Figure S4. Mitochondrial ATP production rate (A) The fundamental parameters of mitochondrial function using the Mito Stress Kit: basal respiration, ATP turnover, proton leak, maximal respiration and spare respiratory capacity. (https://www.agilent.com/en/support/cell-analysis/mitochondrial-respiration-xf-cell-mito-stress-test). (B) Mitochondrial derived ATP production of WT and siPiC HeLa cells with or without treatment was measured using Seahorse, after 48 hours of incubation with siPiC, and another 24 hours incubation with the chimeric protein. ATP levels were reduced by 0.81-fold upon knock down and increased by 1.12-fold when treated with TAT-mPiC protein compared to untreated knock down cells (n=3). Results are presented as mean ± STD. For statistical analysis t test was used (ns P>0.05; * P≤0.05).

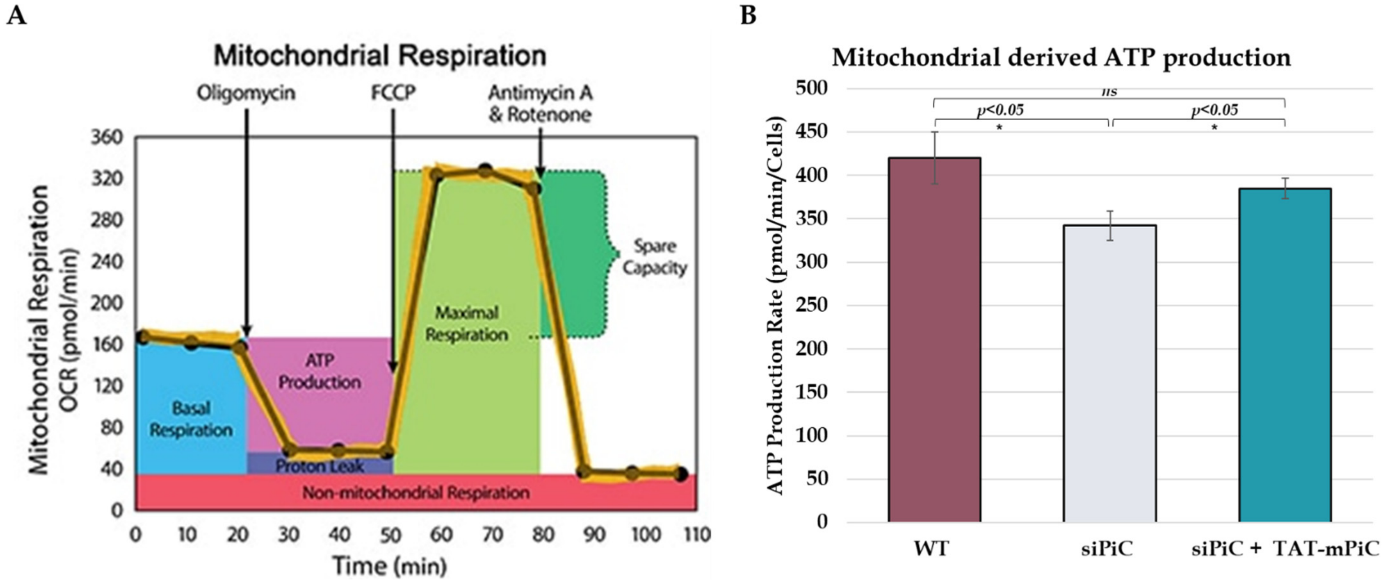

Figure S5. (A) Schematic presentation of the fusion protein, His-TAT-Phosphate carrier variant A-mCherry (His-TAT-mPiC.A_mCherry) MW=70.5 kDa.

Light blue=His-tag for Ni column binding, Bordo=TAT sequence allowing the cargo to cross cell membranes, Mint green=MTS-mitochondrial targeting sequence allowing the protein to localize into mitochondria, Turquoise=the functional human mitochondrial Phosphate Carrier (mPiC) protein, Red= Red fluorescent protein (mCherry).

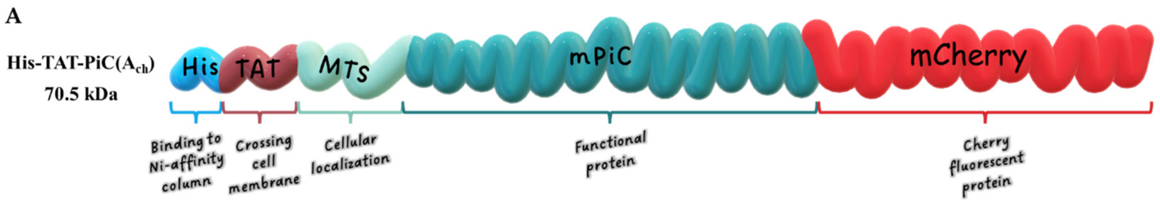

Figure S6. The nucleotide (A) and amino acid (B) sequences of HIS-TEV-TAT-MTS-PiCA.

Light blue=His-tag, Dark blue= TEV protease recognition sequence, Bordo=TAT sequence, Mint green=MTS-mitochondrial targeting sequence, Turquoise=the functional human mitochondrial Phosphate Carrier (mPiC) protein.

A

ATGGGCAGCAGCCATCATCATCATCATCACAGCAGCGGCCTGGTGCCGCGCGGCAGCCATATGGAGAATCTTTA
CTTTCAGGGGAGGAAGAAGCGGAGACAGCGACGAAGAGGCTCGGATCCATTCTCGTCCGTGGCGCACCTGGCG
CGGGCGAACCCCTTCAACACGCCACATCTGCAGCTGGTGCACGATGGTCTCGGGGACCTCCGCAGCAGCTCCCC
AGGGCCCACGGGCCAGCCCCGCCGCCCTCGCAACCTGGCAGCCGCCGCCGTGGAAGAGCAGTATAGCTGTGACT
ATGGATCTGGCAGATTCTTTATCCTTTGTGGACTTGGAGGAATTATTAGCTGTGGCACAACACATACAGCATTGG
TTCCTCTAGATCTGGTTAAATGCAGAATGCAGGTGGACCCCCAAAAGTACAAGGGCATATTTAACGGATTCTCA
GTTACACTTAAAGAGGATGGTGTTCGTGGTTTGGCTAAAGGATGGGCTCCGACTTTCCTTGGCTACTCCATGCAG
GGACTCTGCAAGTTTGGCTTTTATGAAGTCTTTAAAGTCTTGTATAGCAATATGCTTGGAGAGGAGAATACTTAT
CTCTGGCGCACATCACTATATTTGGCTGCCTCTGCCAGTGCTGAATTCTTTGCTGACATTGCCCTGGCTCCTATGG
AAGCTGCTAAGGTTCGAATTCAAACCCAGCCAGGTTATGCCAACACTTTGAGGGATGCAGCTCCCAAAATGTAT
AAGGAAGAAGGCCTAAAAGCATTCTACAAGGGGGTTGCTCCTCTCTGGATGAGACAGATACCATACACCATGAT
GAAGTTCGCCTGCTTTGAACGTACTGTTGAAGCACTGTACAAGTTTGTGGTTCCTAAGCCCCGCAGTGAATGTTC
AAAGCCAGAGCAGCTGGTTGTAACATTTGTAGCAGGTTACATAGCTGGAGTCTTTTGTGCAATTGTTTCTCACCC
TGCTGATTCTGTGGTATCTGTGTTGAATAAAGAAAAAGGTAGCAGTGCTTCTCTGGTCCTCAAGAGACTTGGATT
TAAAGGTGTATGGAAGGGACTGTTTGCCCGTATCATCATGATTGGTACCCTGACTGCACTACAGTGGTTTATCTA
TGACTCCGTGAAGGTCTACTTCAGACTTCCTCGCCCTCCTCCACCCGAGATGCCAGAGTCTCTGAAGAAGAAGCT
TGGGTTAACTCAGTAG

B

MGSSHHHHHHSSGLVPRGSHMENLYFQGRKKRRQRRRGSDPFSSVAHLARANPFNTPHLQLVHDGLGDLRSSSPGPT
GQPRRPRNLAAAAVEEQYSCDYGSGRFFILCGLGGIISCGTTHTALVPLDLVKCRMQVDPQKYKGIFNGFSVTLKEDG
VRGLAKGWAPTFLGYSMQGLCKFGFYEVFKVLYSNMLGEENTYLWRTSLYLAASASAEFFADIALAPMEAAKVRIQ
TQPGYANTLRDAAPKMYKEEGLKAFYKGVAPLWMRQIPYTMMKFACFERTVEALYKFVVPKPRSECSKPEQLVVTF
VAGYIAGVFCAIVSHPADSVVSVLNKEKGSSASLVLKRLGFKGVWKGLFARIIMIGTLTALQWFIYDSVKVYFRLPRP
PPPEMPESLKKKLGLTQ*

Table S1. MPCD patients reported cases, describing the three SLC25A3 mutations.

Table 1
All reported cases of pathologic SLC25A3 variants.

	Family 1			Family 2		Family 3	Family 4
	Patient 1 – 1	Patient 1 – 2	Patient 1 – 3	Patient 2 – 1	Patient 2 – 2	Patient 3 – 1	Patient 4 – 1
Reference	Mayr et al., 2011	Mayr et al. 2011	Mayr et al., 2011	Mayr et al. 2007	Mayr et al., 2007	Bhoj et al., 2014	Bhoj et al. 2014
Ethnicity	Turkish	Turkish	Turkish	Turkish	Turkish	Guatemalan	Haitian/Dominican
Gender	Female	Male	Male	Female	Female	Male	Male
Current age	Died at 6 months	9 years, stable	17 years, stable	Died at 4 months	Died at 9 months	12 months, stable	10 months, stable
SLC25A3 variant, affected exon	Homozygous c.158-9A > G, exon 3A	Homozygous c.158-9A > G, exon 3A	Homozygous c.158-9A > G, exon 3A	Homozygous c.215G > A, exon 3A	Homozygous c.215G > A, exon 3A	Homozygous c.158-9A > G, exon 3A	Compound heterozygous c.599T > G (exon 4), c.886-898delins7 (exon 6)
Affected PiC isoform	A	A	A	A	A	A	A + B
Condition at birth, and onset of symptoms	Information not available	Prenatal hypertrophic cardiomyopathy. Gestation: born at term. After birth: artificial ventilation was required	Gestation: born at term. Birth weight: 3000 g, Apgar score of 3 at 1 min and 9 at 5 min. At 1.5 h: asphyxia resuscitation and artificial ventilation	Gestation: 38 wks. Birth weight: 2600 g (10%), Apgar score of 9 at 1 min and 10 at 5 min. At 12 h: cyanosis and muscular hypotonia	Gestation: born at term. Birth weight: 2870 g (10%), Apgar score of 8 at 1 min and 10 at 5 min, At 10 h: muscular hypotonia, metabolic and lactic acidosis	Gestation: 41 wks. Birth weight: 3990 g (75%), Apgar score of 7 at 1 min and 8 at 5 min. Within 2 h: muscular hypotonia, hypoxia followed rapidly by respiratory failure	Prenatal hypertrophic cardiomyopathy. Gestation: born at term. Birth weight: 2849 g (50%). Apgar score of 8 at 1 min and 9 at 5 min. At 4 wks: worsening heart failure and respiratory status
Clinical picture:	elevated lactate, neonatal hypertrophic cardiomyopathy, muscular hypotonia, limited information	elevated lactate, neonatal hypertrophic cardiomyopathy, 9 yrs: skeletal myopathy, stable hypertrophic cardiomyopathy	elevated lactate, neonatal hypertrophic cardiomyopathy 17 yrs: skeletal myopathy, stable hypertrophic cardiomyopathy	elevated lactate, neonatal hypertrophic cardiomyopathy leading to death, skeletal myopathy, delayed development.	elevated lactate, neonatal hypertrophic cardiomyopathy leading to death, skeletal myopathy, delayed development	lactic acidosis, neonatal hypertrophic cardiomyopathy moderate skeletal myopathy,	no elevated lactate (lactate/pyruvate ↑) Cardiac transplant at 7 months. no myopathy

E.L. Seifert et al. / Biochemical and Biophysical Research Communications 464 (2015) 369–375
